# Efficacy and Safety of Monoclonal Antibody Against Calcitonin Gene-Related Peptide or Its Receptor for Migraine: A Systematic Review and Network Meta-analysis

**DOI:** 10.3389/fphar.2021.649143

**Published:** 2021-03-25

**Authors:** Xing Wang, Yuqi Chen, Jinlei Song, Chao You

**Affiliations:** ^1^ West China Hospital, Sichuan University, Chengdu, China; ^2^ West China Brain Research Centre, Sichuan University, Chengdu, China

**Keywords:** migraine, calcitonin gene-related peptide, monoclonal antibody, headache, meta-analysis

## Abstract

**Background:** The optimal monoclonal antibody against calcitonin gene-related peptide (CGRP) for adult patients with migraine has yet to be determined. Therefore, we aimed to compare the effectiveness of different monoclonal antibodies against CGRP or its receptor for adult patients with migraine through a network meta-analysis of randomized controlled trials.

**Methods:** We systematically searched the MEDILNE, Embase, ClinicalTrials.gov, and Cochrane Library databases for relevant publications from inception until October 30, 2020. Only randomized clinical trials of adults with migraine that assessed any calcitonin gene-related peptide monoclonal antibody and reported clinical outcomes were included. The primary outcomes were changes in monthly migraine days and treatment-emergent adverse events

**Results:** We initially retrieved 2,070 publications, and ultimately, 18 randomized clinical trials totaling 8,926 patients were included. In terms of efficacy, eptinezumab (MD −1.43, 95% CrI −2.59 to −0.36), erenumab (MD −1.61, 95% CrI −2.40 to −0.84), fremanezumab (MD −2.19, 95% CrI −3.15 to −1.25), and galcanezumab (MD −2.10, 95% CrI −2.76 to −1.45) significantly reduced MMDs compared with placebo. In terms of safety, only galcanezumab increased the incidences of TEAEs (RR 1.11, 95% CrI 1.01–1.22) and serious adverse events (RR 2.95, 95% CrI 1.41–6.87) compared with placebo.

**Conclusion:** Most drugs performed similarly and were superior to placebo in most of our analyses. Further head-to-head research on different types of CGRP monoclonal antibodies is necessary to validate the present findings.

## Introduction

Migraine is a debilitating neurological disease and is regarded as one of the most common global causes of disease-related disability (Gaul et al., 2011; Messali et al., 2016; MacGregor, 2017). It is reported that up to 15% of people worldwide suffer from migraine (Rastenyte et al., 2017; James et al., 2018). There are several drugs used to treat migraine patients; however, most of the current therapeutic medications, such as amitriptyline, candesartan, flunarizine, topiramate, propranolol, and venlafaxine, are primarily designed to treat diseases other than migraine. Moreover, due to the lack of efficacy and intolerability of these suboptimal drugs, migraine patients often switch, reinitiate, or discontinue ongoing treatments. It has been reported that approximately 68% of migraine patients discontinued preventive treatment within 6 months due to poor tolerability or insufficient benefit (Ford et al., 2017; González-Hernández et al., 2018). Therefore, novel treatments with improved efficacy and tolerability are warranted to offer new opportunities for this group of patients.

Currently, monoclonal antibodies against calcitonin gene-related peptide or its receptor, including eptinezumab, fremanezumab, galcanezumab, and erenumab, are used for the prevention of migraine (Paemeleire and MaassenVanDenBrink, 2018; Raffaelli et al., 2019). Studies with pairwise comparisons have suggested that CGRP monoclonal antibodies reduce monthly migraine days without increasing adverse events compared with placebo (Alasad and Asha, 2020; Deng et al., 2020). However, due to a lack of direct comparison of different kinds of CGRP monoclonal antibodies, the relative safety and efficacy of these drugs have not been investigated in depth.

Network meta-analysis has a unique strength over conventional pairwise meta-analysis to provide a more comprehensive analysis of evidence since the former technique enables different interventions to be assessed both directly and indirectly even in the absence of direct comparisons. Therefore, we conducted a systematic review with network meta-analysis to compare the efficacy and safety of all kinds of CGRP monoclonal antibodies and performed a comprehensive ranking of various medications to determine which medication can efficiently and safely reduce migraine headache days per month.

## Methods

### Guidance and Search Strategy

The study was conducted in accordance with the Cochrane Handbook for Systematic Reviews of Interventions and reported in accordance with the Preferred Reporting Items for Systematic Reviews and Meta-analyses Extension Statement for network Meta-analyses (PRISMA-NMA) guidelines (Hutton et al., 2015). It was registered on the OSF platform (http://osf.io/9b8ew).

We searched the MEDLINE, Cochrane CENTRAL, ClinicalTrials.gov, and embase bibliographic databases from inception until October 30, 2020 (Supplementary Table S1). No language limitations were applied. The following MeSH terms and free-text terms such as “migraine,” “migraine headache,” “calcitonin gene-related peptide binding monoclonal antibody,” “eptinezumab,” “ALD403,” “erenumab,” “AMG334,” “fremanezumab,” “TEV-48125,” “galcanezumab,” “LY2951742,” and “randomized controlled trial” were used to identify any eligible publications. Additional studies were identified by searching previous systematic reviews and meta-analyses and by searching the reference lists of the included trials.

### Inclusion and Exclusion Criteria

We included adult patients (more than 18 years old) who were diagnosed with migraine according to the International Classification of Headache Disorders second edition (ICHD-II) (Olesen, 2005), or the third edition (ICHD-Ⅲ, beta version) (Headache Classification Committee of the International Headache Society (IHS), 2013). We defined intervention as the use of any type of monoclonal antibody against calcitonin gene-related peptide or its receptor at commercial doses, (i.e. 120 mg of galcanezumab (LY2951742), 70 mg of erenumab (AMG334), 100 mg of eptinezumab (ALD403), or 225 mg of fremanezumab (TEV-48125). The control group was defined as placebo or different types of CGRP monoclonal antibodies. We chose changes in the number of monthly migraine days from baseline to endpoint as the primary efficacy outcome measure and the proportion of participants who suffered treatment-emergent adverse events (TEAEs) as the primary safety outcome measure. Additionally, the frequency of patients with at least 50 and 75% reductions in the days with migraine (50 and 75% response rates) as well as the proportion of participants who suffered serious adverse events were assessed as secondary outcomes. We only included randomized controlled trials and excluded observational or cross-sectional studies.

Additionally, we excluded 1) trials that only compared different doses of a single CGRP monoclonal antibody, 2) trials that only compared a CGRP monoclonal antibody with other pharmacologically active drugs, and 3) trials that assessed calcitonin gene-related peptide binding monoclonal antibodies in pediatric migraine patients.

### Selection Process and Data Extraction

After deleting duplicates, two reviewers manually filtered articles that were deemed ineligible by screening titles and abstracts. Then, the full texts of articles were reviewed and further screened based on the eligibility criteria mentioned above. Two reviewers independently completed this procedure together. Conflicts in study selection were resolved by comprehensive discussion or by consulting a third independent reviewer for help.

The following data were extracted onto a modified table form of the data extraction template designed by the Cochrane Public Health Group: study characteristics such as primary author, year of publication, geographical location, numbers of centers that the study included, duration of follow-up, etc.; patient characteristics such as age, sex, condition of disease, etc.; and treatment characteristics such as type and dose of the drugs. Two reviewers worked in pairs to extract data from the eligible studies. In cases of incomplete or ambiguous data, we contacted the corresponding author of the article or the editor of the journal. Disagreements on data extraction were settled by comprehensive discussion or turned to a third independent reviewer for help.

### Assessment of Risk of Bias and Quality of Evidence

The same two reviewers evaluated the risk of bias of each trial by using the tool designed by the Cochrane Statistical Methods Group across seven domains: allocation concealment, incomplete outcome data, blinding of study participants, random sequence generation, selective reporting, blinding of outcome assessment, and other potential risk of bias (Higgins et al., 2011). The risk of bias in each domain was assessed as either low, unclear, or high. A trial was rated as having an overall low risk of bias if each domain was assessed to have a low risk of bias. Otherwise, it was judged as having an overall high risk of bias. We contacted the original study investigators for more information if necessary.

Additionally, the certainty of evidence of the primary outcomes was assessed using a framework developed by the GRADE working group: Grading of Recommendations Assessment, Development, and Evaluation for rating the certainty of effect estimates (Atkins et al., 2004). A total of five domains were evaluated: limitations in design, indirectness, imprecision, publication bias, and inconsistency. The overall quality of evidence was further rated “high,” “moderate,” “low,” or “very low.”

### Statistical Analysis

We conducted a Bayesian, random effects, consistency, network meta-analysis in R software (gemtc package) to incorporate indirect comparisons. We further modeled the comparative efficacy and safety of any two different drugs as a function of each drug relative to another drug. The Markov chain Monte Carlo model was set to have 20,000 simulated draws after a burn-in of 8,000 iterations. The point estimates [relative risk (RRs)] and the corresponding 95% CrIs (credible intervals) were obtained by the 2.5th and 97.fifth percentiles of the final posterior distribution. The probability of each medication being at each possible rank was also estimated to rank the intervention hierarchy in the network meta-analysis by using the rankogram function in R software. For continuous variables that provided inexhaustive results, we used the formula suggested by the Cochrane Handbook for Systematic Reviews of Interventions. Potential publication bias was evaluated by the funnel plot, and we used Egger's regression test and Begg's adjusted rank correlation test to assess asymmetry of the funnel plot if ten or more studies were included. Furthermore, we conducted sensitivity analysis for primary and secondary outcomes by including phase III trials only.

All analyses were performed in R (release version 4.0.3) and RevMan (5.4.1; The Cochrane Collaboration). A two-sided *p* value of less than 0.05 was regarded as statistically significant.

## Results

### Search Results

**FIGURE 1:**
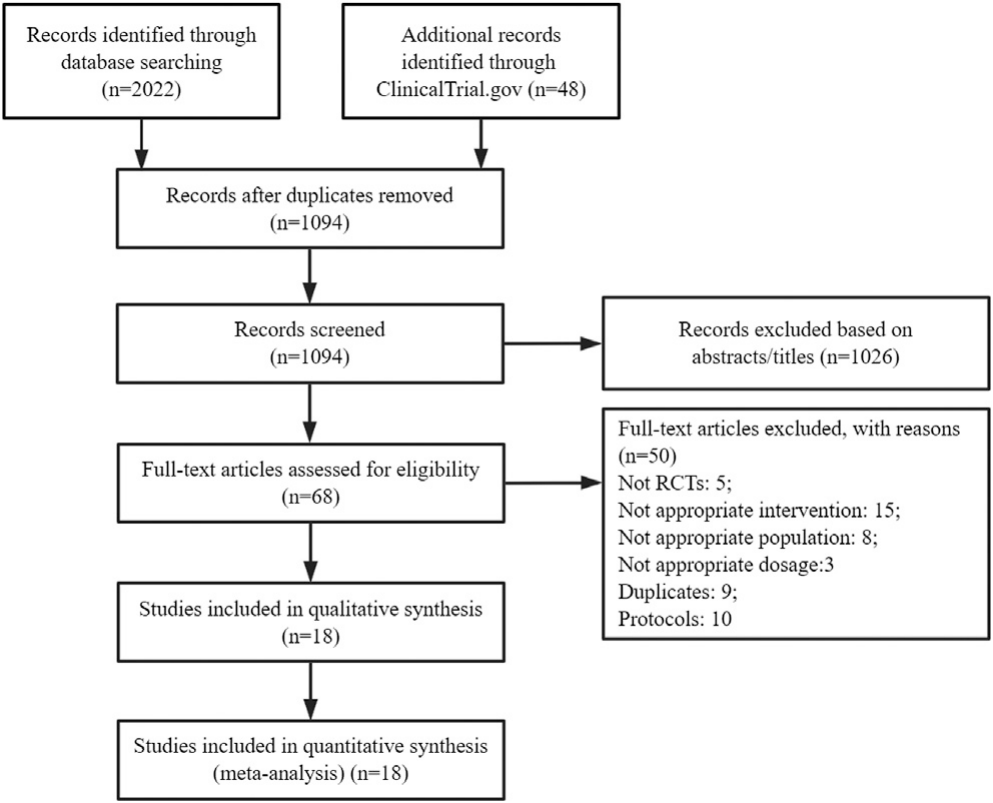
PRISMA flow diagram showing the process of study selection. RCT: Randomized controlled trials.

We initially identified 2,070 potentially relevant articles after searching several databases. Then, 1,094 articles were screened after removing duplicates and ineligible studies by scanning titles and/or abstracts. The full texts of sixty-eight articles were assessed. Among them, 50 studies were deemed ineligible for the reasons listed in Figure 1. Finally, 18 studies were included in the systematic review with network meta-analysis (Bigal et al., 2015; Goadsby et al., 2017; Sun et al., 2016; Silberstein et al., 2017; Tepper et al., 2017; Detke et al., 2018; Dodick et al., 2018a; Dodick et al., 2018b; Skljarevski et al., 2018a; Skljarevski et al., 2018b; Stauffer et al., 2018; Dodick et al., 2019; Ferrari et al., 2019; Sakai et al., 2019; Ashina et al., 2020; Lipton et al., 2020; Mulleners et al., 2020; NCT02959177, 2020).

### Study Characteristics

Overall, 18 trials totaling 8,926 patients were deemed eligible and included. Three trials assessed the effects of eptinezumab; five trials assessed erenumab; four trials assessed fremanezumab; and six trials assessed galcanezumab. The sample sizes in each trial ranged from 200 to 836, and the mean sample size was 496. The median mean age in the control group of the included trials was 41.8 years. All of the included trials mostly enrolled female patients, and the median proportion of females in the control group was 85.7%. Participants were followed up for 12 weeks in the majority of eligible trials. Only five trials (27.8%) completed follow-up visits until 24 weeks. Thirteen studies (72.2%) were conducted in multiple countries, and all studies were multicenter trials. Seven studies were phase 2 trials, and eleven studies were phase 3 trials. Table 1 summarizes the study characteristics.

**TABLE 1:** Characteristics of trials included in the systematic review and network meta-analysis.

Trial	Registration number	Trial characteristic	Country (centers)	No. of patients	Intervention		Control		Primary outcomes	Follow up
					Protocol	Age (% female)	Protocol	Age (% female)		
Dodick 2019	NCT02275117	Phase 2	4 countries (92)	243	100 mg Eptinezumab	36.7 (85%)	Placebo	37.2 (90%)	75% response rates	12 weeks
PROMISE-1 2020	NCT02559895	Phase 3	2 countries (84)	445	100 mg Eptinezumab	40.0 (80%)	Placebo	39.9 (84%)	Change in MMDs	12 weeks
PROMISE-2 2020	NCT02974153	Phase 3	13 countries (128)	722	100 mg Eptinezumab	41.0 (86%)	Placebo	39.6 (89%)	Change in MMDs	12 weeks
Sun 2016	NCT01952574	Phase 2	7 countries (59)	267	70 mg Erenumab	42.6 (77%)	Placebo	41.4 (83%)	Change in MMDs	12 weeks
STRIVE 2017	NCT02456740	Phase 3	Multiple countries (121)	636	70 mg Erenumab	41.1 (84.5%)	Placebo	41.3 (85.9%)	Change in MMDs	24 weeks
Tepper 2017	NCT02066415	Phase 2	10 countries (69)	477	70 mg Erenumab	41.4 (87%)	Placebo	42.1 (79%)	Change in MMDs	12 weeks
ARISE 2018	NCT02483585	Phase 3	Multiple countries (69)	577	70 mg Erenumab	42 (85.7%)	Placebo	42 (84.9%)	Change in MMDs	12 weeks
Sakai 2019	NCT02630459	Phase 2	Japan (43)	271	70 mg Erenumab	44 (85.2%)	Placebo	45 (86.8%)	Change in MMDs	24 weeks
Bigal 2015	NCT02025556	Phase 2	United States (62)	200	225 mg Fremanezumab	40.8 (91%)	Placebo	42.0 (88%)	Change in MMDs	12 weeks
Silberstein 2017	NCT02621931	Phase 3	9 countries (132)	754	225 mg Fremanezumab	40.6 (87%)	Placebo	41.4 (88%)	Change in MHDs	12 weeks
Dodick 2018	NCT02629861	Phase 3	9 countries (123)	584	225 mg Fremanezumab	42.9 (84.1%)	Placebo	41.3 (84.0%)	Change in MMDs	12 weeks
FOCUS 2019	NCT03308968	Phase 3	14 countries (104)	562	225 mg Fremanezumab	45.9 (84%)	Placebo	46.8 (84%)	Change in MMDs	12 weeks
EVOLVE-1 2018	NCT02614183	Phase 3	United States (90)	646	120 mg Galcanezumab	40.9 (85%)	Placebo	41.3 (83.6%)	Change in MMDs	24 weeks
EVOLVE-2 2018	NCT02614196	Phase 3	11 countries (109)	692	120 mg Galcanezumab	40.9 (85.3%)	Placebo	42.3 (85.7%)	Change in MMDs	24 weeks
REGAIN 2018	NCT02614261	Phase 3	12 countries (116)	836	120 mg Galcanezumab	39.7 (85%)	Placebo	41.6 (87%)	Change in MMDs	12 weeks
Skljarevski 2018	NCT02163993	Phase 2	United States (multiple centers)	207	120 mg Galcanezumab	NA	Placebo	39.5 (79.6%)	Change in MMDs	12 weeks
CONQUER 2020	NCT03559257	Phase 3	12 countries (64)	462	120 mg Galcanezumab	45.9 (84%)	Placebo	45.7 (88%)	Change in MMDs	12 weeks
NCT02959177	NCT02959177	Phase 2	Japan (47)	345	120 mg Galcanezumab	NA	Placebo	NA	Change in MMDs	24 weeks

This data extracted from control group since data of all patients lacked. MMD: monthly migraine days; MHD: monthly headache days; USA: United States of America; NA: not applicable.

### Primary Outcomes

**FIGURE 2:**
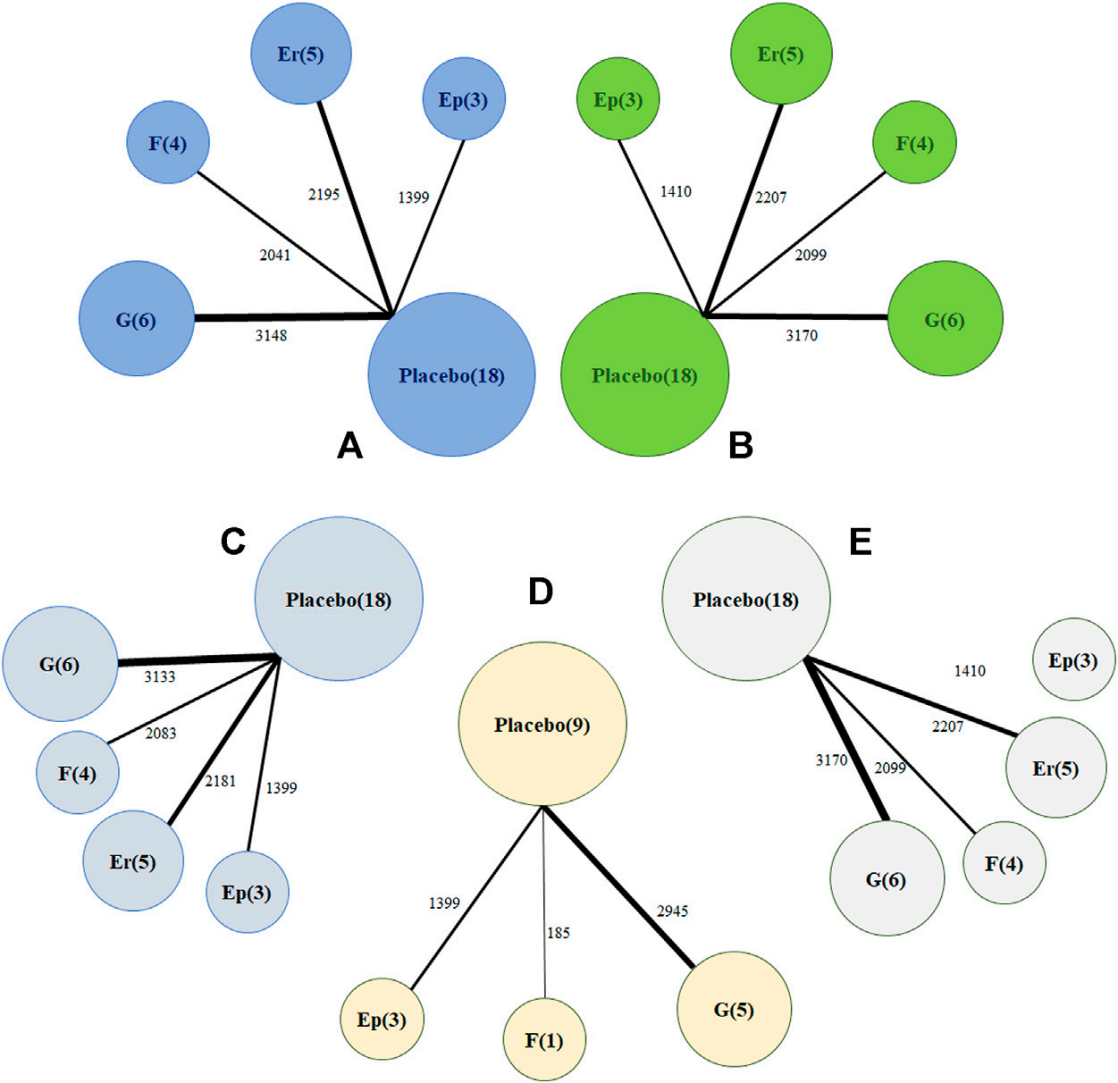
Network plot of **(A)** change in monthly migraine days **(B)** treatment-emerging adverse events **(C)** 50% response rates **(D)** 75% response rates **(E)** serious adverse events. The width of the lines is proportional to the number of studies comparing every pair of treatments, and the size of each circle is proportional to the number of participants. Ep: eptinezumab; Er: erenumab; **(F)**: fremanezumab; **(G)**: galcanezumab.

**FIGURE 3:**
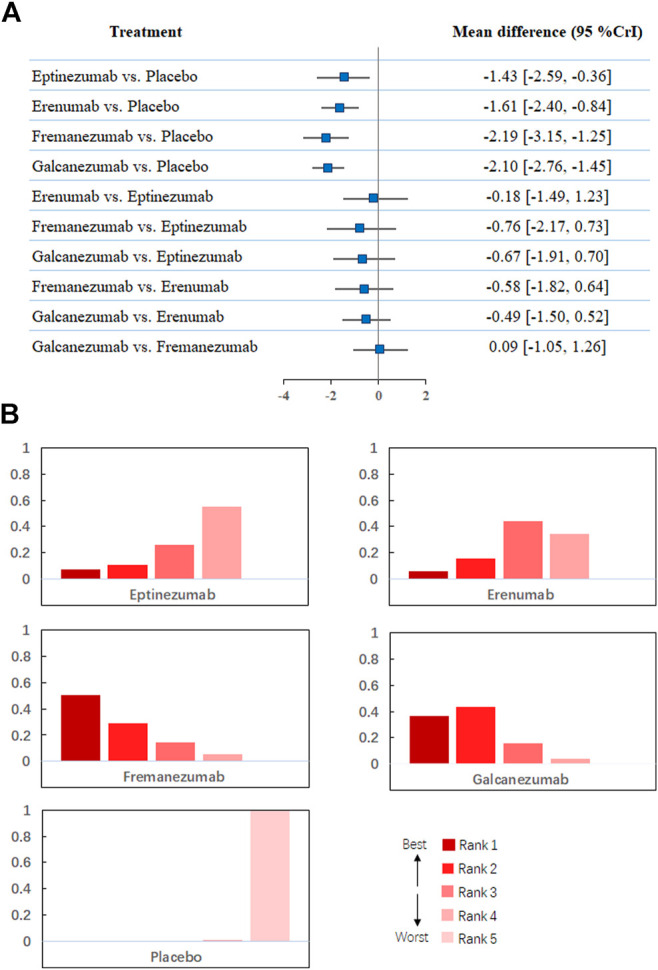
**(A)**. Network meta-analysis of change in monthly migraine days. **(B)**. Ranking positions of different drugs in monthly migraine days. CrI: credible interval.

Regarding changes from baseline in monthly migraine headaches, a network of comparisons of different types of CGRP monoclonal antibodies was reported in all included trials, totaling 8,783 participants (Figure 2A). Figure 3 presents the pooled estimates of the results from the network meta-analysis for the efficacy of CGRP monoclonal antibodies. Among all the treatments, fremanezumab had the highest probability of being ranked first to reduce monthly migraine days (MD −2.19, 95% CrI −3.15 to −1.25, compared with placebo), followed by galcanezumab (MD −2.10, 95% CrI −2.76 to −1.45, compared with placebo), erenumab (MD −1.61, 95% CrI −2.40 to −0.84, compared with placebo), and eptinezumab (MD −1.43, 95% CrI −2.59 to −0.36, compared with placebo). Between-drug comparisons did not show significant differences.

**FIGURE 4:**
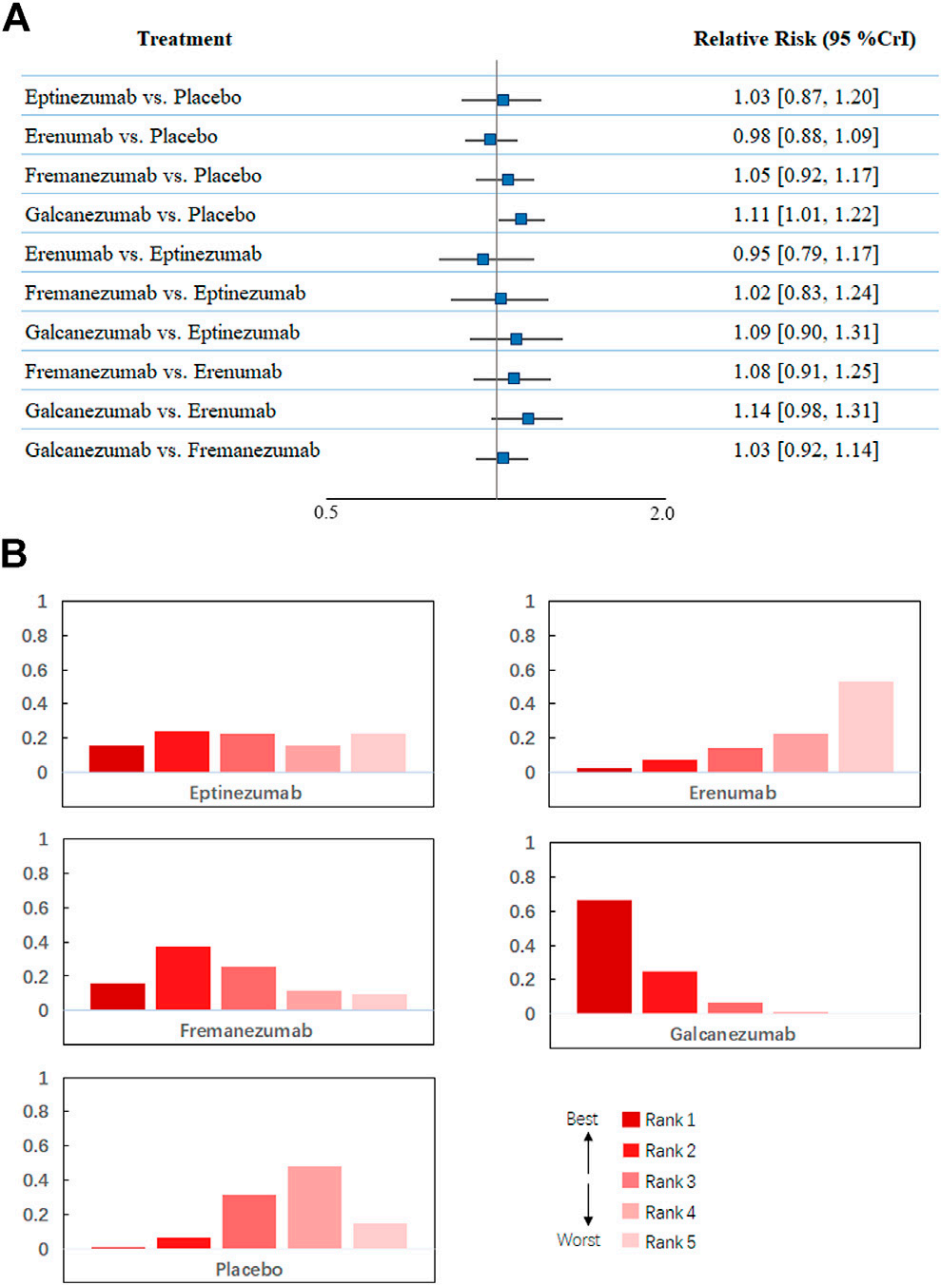
**(A)**. Network meta-analysis of treatment-emerging adverse events. **(B)**. Ranking positions of different drugs in treatment-emerging adverse events. CrI: credible interval.

Regarding treatment-emergent adverse events, Figure 2B shows that all 18 trials comprising 8,886 participants reported this outcome. A total of 2,125 patients (54.5%) developed at least one adverse reaction during or after therapy in the treatment group compared with 2,656 in the placebo group (53.3%). The network meta-analysis demonstrated that galcanezumab was more likely to cause at least one more treatment-emergent adverse event than placebo (RR 1.11, 95% CrI 1.01–1.22) and had the highest probability of being ranked first to increase the incidence of TEAEs (Figure 4), followed by fremanezumab (RR 1.05, 95% CrI 0.92 to 1.17, compared with placebo), eptinezumab (RR 1.03, 95% CrI 0.87 to 1.20, compared with placebo), and erenumab (RR 0.98, 95% CrI 0.88 to 1.09, compared with placebo). According to the majority of the included studies, the most common adverse reactions related to galcanezumab were injection site pain, upper respiratory tract infection, and nasopharyngitis.

### Secondary Outcomes

In the network meta-analysis of at least 50% response rates, 18 trials comprising 8,796 patients were pooled (Figure 2C). Most treatments, including erenumab (RR 1.75, 95% CrI 1.30–2.44), fremanezumab (RR 2.29, 95% CrI 1.63–3.25), and galcanezumab (RR 1.53, 95% CrI 1.18–1.99), were significantly more effective than placebo (Table 2). Our analysis also demonstrated that fremanezumab was superior to eptinezumab (RR 1.65, 95% CrI 1.00–2.75) in reducing the frequency of headache attacks by at least 50%. According to the rankograms, fremanezumab had the highest probability of being ranked best, followed by erenumab, galcanezumab, and eptinezumab (Supplementary Figure S1). In terms of at least 75% response rates, we summarized the results of nine trials totaling 4,529 patients (Figure 2D). The network meta-analysis showed that all of the available drugs, including eptinezumab (RR 1.59, 95% CrI 1.15–2.20), fremanezumab (RR 3.23, 95% CrI 1.52–7.36), and galcanezumab (RR 2.14, 95% CrI 1.69–2.95), were more effective in reducing the frequency of headache attacks by at least 75% compared with placebo. Fremanezumab had the highest probability of being ranked best, followed by galcanezumab and eptinezumab (Supplementary Figure S2).

**TABLE 2:** Pooled RR and relative CrI of 50% response rates, 75% response rates, and serious adverse events derived from network meta-analysis with different treatment regimens in patients with migraine.

Intervention	50% response rates	75% response rates	Serious adverse events
Compared with placebo			
Eptinezumab	1.40 [0.97, 2.02]	1.59 [1.15, 2.20]	1.25 [0.49, 3.35]
Erenumab	1.75 [1.30, 2.44]	–	1.15 [0.57, 2.39]
Fremanezumab	2.29 [1.63, 3.25]	3.23 [1.52, 7.36]	1.16 [0.52, 2.88]
Galcanezumab	1.53 [1.18, 1.99]	2.14 [1.69, 2.95]	2.95 [1.41, 6.87]
Compared with eptinezumab			
Erenumab	1.25 [0.78, 2.07]	–	0.91 [0.27, 3.02]
Fremanezumab	1.65 [1.00, 2.75]	2.04 [0.89, 4.96]	0.94 [0.27, 3.42]
Galcanezumab	1.09 [0.70, 1.73]	1.35 [0.91, 2.15]	2.38 [0.70, 8.65]
Compared with erenumab			
Fremanezumab	1.30 [0.82, 2.05]	–	1.00 [0.34, 3.25]
Galcanezumab	0.88 [0.58, 1.29]	–	2.56 [0.93, 7.84]
Compared with fremanezumab			
Galcanezumab	0.67 [0.43, 1.03]	0.67 [0.28, 1.52]	2.54 [0.80, 7.95]

RR: relative risk; CrI: credibility interval.

In terms of serious adverse events, a total of 18 trials with 8,886 patients reported this outcome (Figure 2E). Our analysis found that only galcanezumab increased the risk of serious adverse events compared with placebo (RR 2.95, 95% CrI 1.41–6.87). Galcanezumab ranked highest for causing at least one serious adverse event, followed by eptinezumab, erenumab, and fremanezumab (Supplementary Figure S3). We also extracted relevant information on serious adverse events in each trial, as shown in Supplementary Table S2. The serious adverse events reported by different trials varied greatly; however, few of them were related to the study drugs.

### Risk of Bias Judgment and Certainty of Evidence Assessment

All included trials used adequate methods to generate random sequences and conceal allocation. Fifteen trials were judged as having an overall low risk of bias. The other three trials were regarded as having an unclear risk of bias. Two trials were judged as having an unclear risk of bias due to selective reporting bias; the other trial was judged as having an unclear risk of bias due to selective reporting bias and other biases. Supplementary Figures S4–S5 present the full details of the risk of bias assessment for each study. The quality of the evidence for primary outcomes is summarized in Supplementary Table S3. In general, the certainty of evidence for each estimate was judged to be moderate to high.

## Discussion

To our knowledge, there is currently no direct comparison of the different monoclonal antibodies against CGRP for migraine in adults. Therefore, network meta-analysis could provide clinicians and researchers with comparative effectiveness of these novel therapeutic drugs. Our network meta-analysis focused on four monoclonal antibodies against CGRP involving 8,926 patients by pooling data derived from 18 RCTs. Pooled results showed that all of the drugs were reported to be similarly effective in reducing monthly migraine days. However, galcanezumab was found to be more likely to cause TEAEs and serious adverse reactions compared with placebo. In addition, we found that fremanezumab provides an advantage over eptinezumab in terms of improving response rates by at least 50%. However, the difference was too small to draw a firm conclusion. Most drugs performed similarly and were superior to placebo in most of our analyses. Our findings call for future head-to-head studies to examine the associations between these medications in migraine therapy among adult patients. It is necessary to point out that erenumab is a monoclonal antibody against the CGRP receptor complex, while eptinezumab, fremanezumab, and galcanezumab are monoclonal antibodies against CGRP (Deen et al., 2017; Edvinsson, 2017). Current studies have shown that some of these novel drugs are effective and well tolerated in the long term (Ashina et al., 2021); however, attention should be devoted to the potential risks of causing hypertension and worsening ischemic stroke reported by the latest research (Russell et al., 2014; Mulder et al., 2020; Saely et al., 2021).

### Comparison With Other Studies

From 2017 to 2020, several researchers conducted meta-analyses to evaluate treatment with GCRP monoclonal antibody in migraine patients (Hou et al., 2017; Zhu et al., 2018; Han et al., 2019; Xu et al., 2019; Alasad and Asha, 2020; Deng et al., 2020). Though the meta-analyses were slightly different in design, they all used direct methods to compare the efficacy and safety of the medication with placebo. Their results remained similar in that CGRP-binding monoclonal antibodies significantly reduced the monthly migraine days without increasing the incidence of adverse events. Although they performed subgroup analyses of different types of GCRP monoclonal antibodies, the subgroup analyses did not show any significant difference. Their conclusions might be limited since most of the previous studies did not perform comprehensive literature searches and failed to show the comparative effectiveness of various types of CGRP monoclonal antibodies.

### Strengths and Limitations of the Study

Network meta-analysis allowed us to compare medication classes with placebo both directly and indirectly, which usually provides a more precise estimate of the relative efficacy and safety than pairwise analyses. A key strength of our study is the use of a network method to explore the relative effect of different types of CGRP monoclonal antibodies for migraine. By using network analysis, we were able to incorporate direct and indirect comparisons among various types of CGRP monoclonal antibodies to rank their efficacy and safety for migraine therapy. In addition, we reasonably used the GRADE method to judge the certainty of evidence for the primary estimates. These methods were useful for clinical decision making. We also provided more up-to-date information regarding the reported efficacy and safety of CGRP monoclonal binding antibody in treating adult patients with migraine. Indeed, this meta-analysis covered a greater number of recent trials, including the PROMISE-1 and CONQUER trials as well as the unpublished NCT02959177 trial.

There are several limitations related to our study. First, the inclusion criteria varied in different trials. Some trials only included patients with episodic migraine, some included patients with chronic migraine, and some mixed these two subtypes of disease.

Second, several trials did not provide treatment-emerging adverse events; therefore, data on adverse events were used instead.

Third, our network meta-analysis assessed different drugs at commercial doses. Whether drugs at other doses provide better effects remains to be explored.

Fourth, the present analysis showed that fremanezumab displayed better efficacy in improving 50% response rates than eptinezumab; however, the difference was minor (95% CrI: 1.00, 2.75). Further head-to-head studies are warranted to validate this finding.

Fifth, we could not explore the cost-effectiveness of these drugs due to a lack of relevant data. Further studies should pay attention to this problem and compare cost-effectiveness with other available drugs.

### Implications in Practice

European Headache Federation (EHF) Guidelines for migraine management recommend the use of several monoclonal antibodies against calcitonin gene-related peptides or its receptors, including eptinezumab, erenumab, fremanezumab, and galcanezumab. However, they did not provide any advice on which drug was the most effective. Our findings suggest that 225 mg fremanezumab might be more effective in reducing MMDs and 120 mg galcanezumab might increase the incidence of TEAEs and serious adverse events in treating people with migraine. These findings are useful for guideline development and helping clinicians to make decisions as to which drug to use in the absence of head-to-head trials. However, further head-to-head studies are needed to testify the present findings. The cost of all of these drugs relative to other treatments for migraine is also a factor that needs to be taken into account. Clinicians should make comprehensive considerations based on efficacy, safety, and cost-effectiveness.

## Conclusion

In summary, most drugs performed similarly in efficacy and safety profile and were superior to placebo according to most of our analyses. Our analysis also suggests that fremanezumab might offer the first level in terms of reducing monthly migraine days and galcanezumab might rank the highest in terms of causing TEAEs and serious adverse events in patients with migraine. Further research on various types of CGRP monoclonal antibodies is needed to validate the present findings.

## Data Availability

The original contributions presented in the study are included in the article/Supplementary Material, further inquiries can be directed to the corresponding author.
