# Supplementary material for: Efficacy and Safety of Monoclonal Antibody Against Calcitonin Gene-Related Peptide or Its Receptor for Migraine: A Systematic Review and Network Meta-analysis
Source: Front Pharmacol. 2021 Mar 25;12:649143. doi: 10.3389/fphar.2021.649143 (PMC8045977; doi:10.3389/fphar.2021.649143)
Supplement: Supplementary file 1 [file Table1.docx]

### Table A1: Search Strategy

| OVID EMBASE (adapted for other databases) | |
| --- | --- |
| 1 | exp Migraine Disorders/ |
| 2 | (Migraine* OR Migraine Headache* OR Sick Headache* OR Disorder*, Migraine* OR Headache*, Migraine*).ab,kw,ti |
| 3 | 1 or 2 |
| 4 | (eptinezumab OR ALD403).mp |
| 5 | (Galcanezumab OR LY2951742).mp |
| 6 | (erenumab OR AMG334).mp |
| 7 | (fremanezumab OR TEV-48125).mp |
| 8 | 4 or 5 or 6 or 7 |
| 9 | 3 and 8 |
| 10 | randomized controlled trial.pt. |
| 11 | controlled clinical trial.pt. |
| 12 | (randomly OR randomized OR placebo OR trial OR groups).ab,kw,ti |
| 13 | 10 or 11 or 12 |
| 14 | exp animals/ |
| 15 | exp human/ |
| 16 | 14 not 15 |
| 17 | 13 not 16 |
| 18 | 9 and 17 |
