# Supplementary figures and images for: Efficacy and Safety of Monoclonal Antibody Against Calcitonin Gene-Related Peptide or Its Receptor for Migraine: A Systematic Review and Network Meta-analysis
Source: Front Pharmacol. 2021 Mar 25;12:649143. doi: 10.3389/fphar.2021.649143 (PMC8045977; doi:10.3389/fphar.2021.649143)

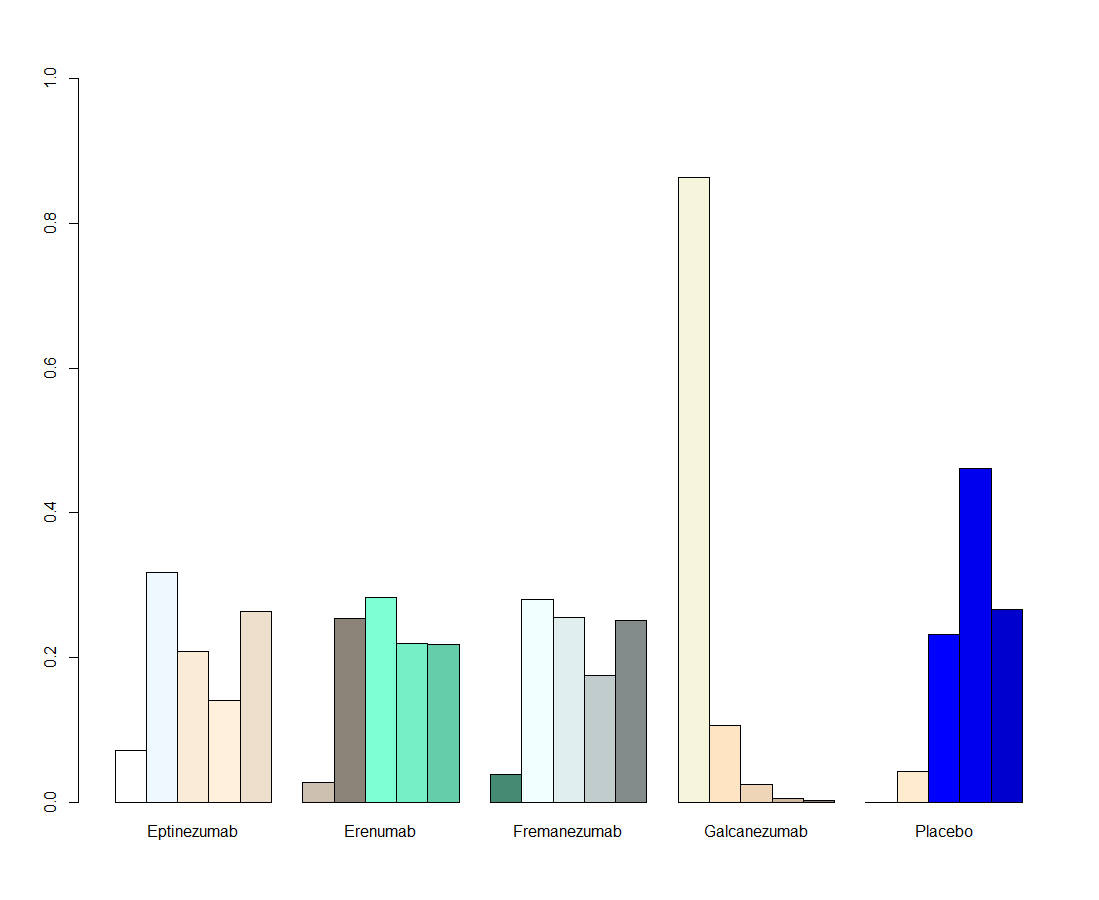

Supplement: Supplementary file 2 [file Image3.tif]

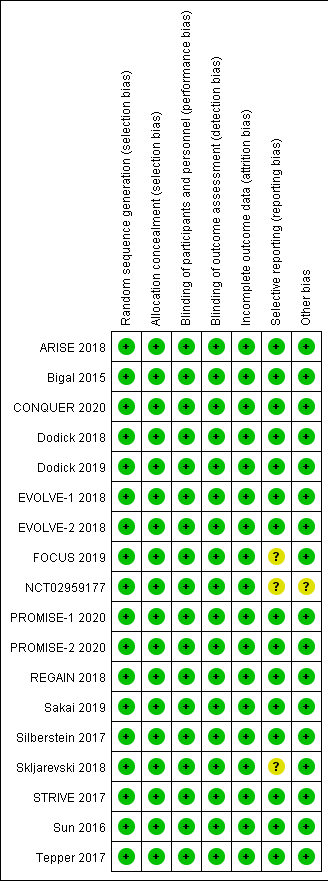

Supplement: Supplementary file 3 [file Image4.tif]

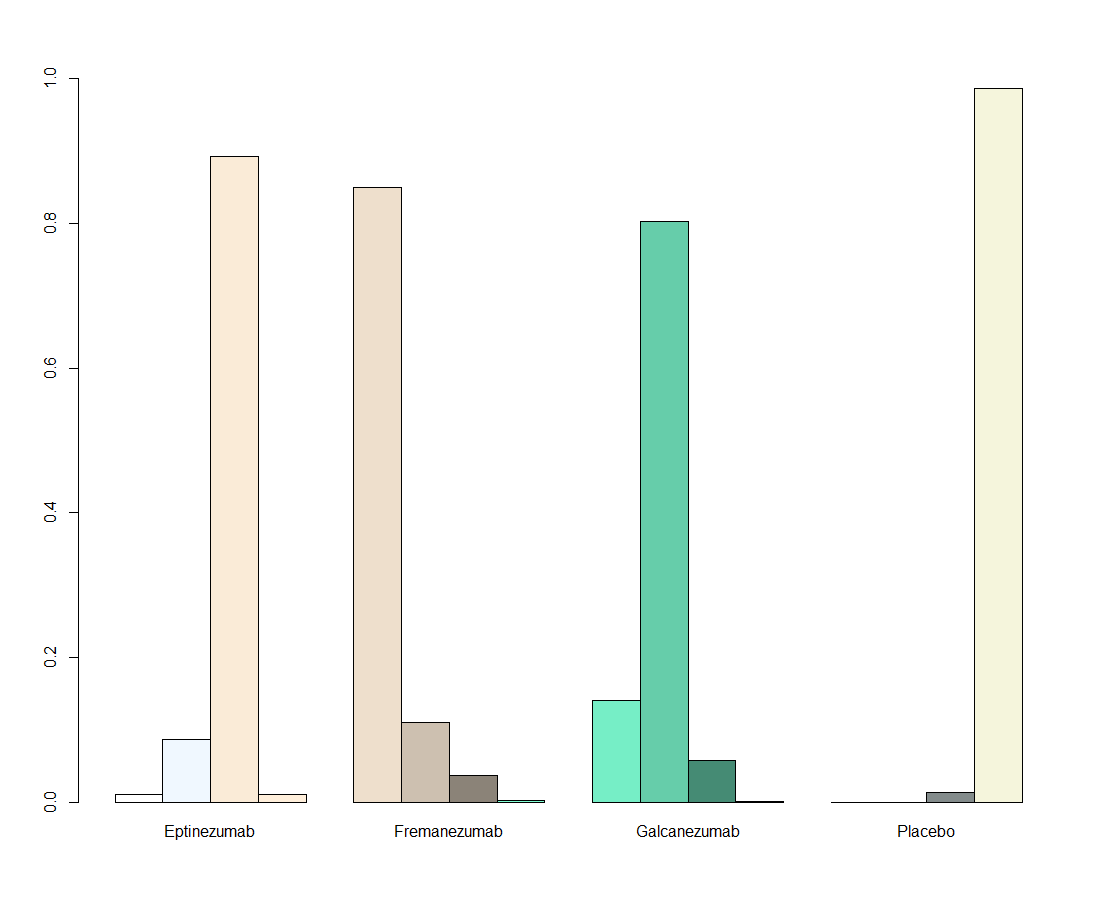

Supplement: Supplementary file 4 [file Image2.tif]

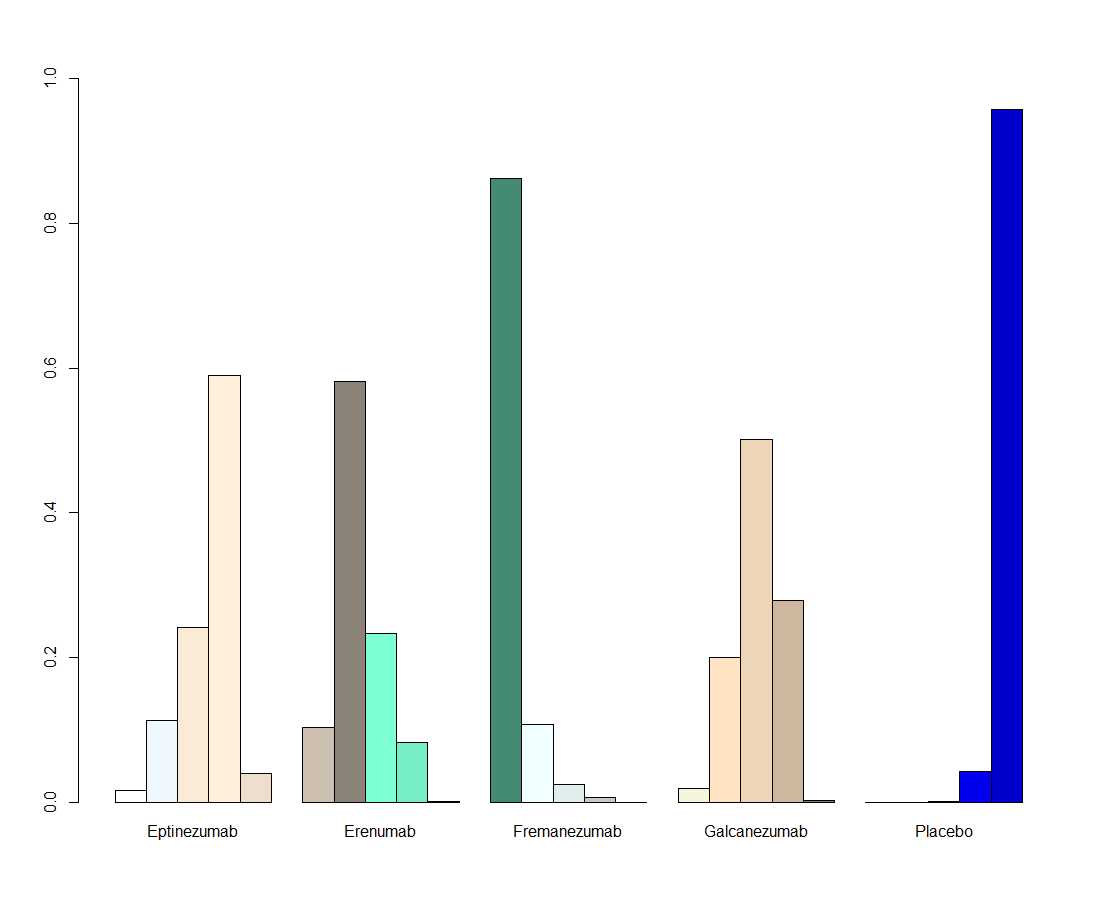

Supplement: Supplementary file 5 [file Image1.tif]

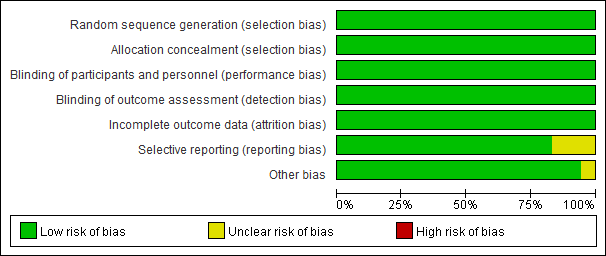

Supplement: Supplementary file 9 [file Image5.tif]
