# Supplementary material for: Efficacy and Safety of Monoclonal Antibody Against Calcitonin Gene-Related Peptide or Its Receptor for Migraine: A Systematic Review and Network Meta-analysis
Source: Front Pharmacol. 2021 Mar 25;12:649143. doi: 10.3389/fphar.2021.649143 (PMC8045977; doi:10.3389/fphar.2021.649143)
Supplement: Supplementary file 6 [file Table2.docx]

| Trial | Intervention | Serious adverse events |
| --- | --- | --- |
| Dodick 2019 | 100mg Eptinezumab | Neezing, cough, nasal congestion, scratchy throat, burning and watery eyes, edema in the face and eyelids, shortness of breath, hives, and itching; none were considered related to the study drug. |
| PROMISE-1 2020 | 100mg Eptinezumab | Acute kidney injury, stomal hernia, and rhabdomyolysis; no SAEs were considered related to drugs |
| PROMISE-2 2020 | 100mg Eptinezumab | Nervous system disorders, injury, poisoning, procedural complications, and psychiatric disorders |
| Sun 2016 | 70mg Erenumab | Vertigo and migraine |
| STRIVE 2017 | 70mg Erenumab | Noncardiac chest pain, cholelithiasis, back pain, ovarian cyst, migraine, post-traumatic neck syndrome, acute pyelonephritis |
| Tepper 2017 | 70mg Erenumab | Intervertebral disc protrusion, appendicitis, costochondritis, fibroma, non-cardiac chest pain, radius fracture |
| ARISE 2018 | 70mg Erenumab | Migraine, intervertebral disc protrusion, urinary tract infection |
| Sakai 2019 | 70mg Erenumab | Systemic lupus erythematosus, hand fracture, gastroenteritis and intestinal tuberculosis. |
| Bigal 2015 | 225mg Fremanezumab | Fibula fracture, migraine |
| Silberstein 2017 | 225mg Fremanezumab | Injury, poisoning and procedural complications, musculoskeletal and connective tissue disorders, psychiatric disorders, renal and urinary disorders, hypertensive crisis |
| Dodick 2018 | 225mg Fremanezumab | Erythema, injection site induration, diarrhea, anxiety, and depression |
| FOCUS 2019 | 225mg Fremanezumab | Atrial fibrillation, cholelithiasis, clavicle fracture, foot fracture, respiratory fume inhalation, rib fracture, road traffic accident, back pain, nephrolithiasis, and vocal cord thickening |
| EVOLVE-1 2018 | 120mg Galcanezumab | Tubular breast carcinoma, vertebral osteophyte, acute pancreatitis, incarcerated incisional hernia and seroma |
| EVOLVE-2 2018 | 120mg Galcanezumab | adenocarcinoma of the cervix, bladder dysfunction, gastritis, bacterial pharyngitis, and rectal polyp |
| REGAIN 2018 | 120mg Galcanezumab | Colon cancer |
| Skljarevski 2018 | 120mg Galcanezumab | Appendicitis |
| CONQUER 2020 | 120mg Galcanezumab | Hemorrhoids and tonsillitis |
| NCT02959177 | 120mg Galcanezumab | Sudden hearing loss, tooth impacted, meniscus injury |

**Table A2: Serious adverse events of trials included in the systematic review and network meta-analysis**
