# Supplementary material for: Efficacy and Safety of Monoclonal Antibody Against Calcitonin Gene-Related Peptide or Its Receptor for Migraine: A Systematic Review and Network Meta-analysis
Source: Front Pharmacol. 2021 Mar 25;12:649143. doi: 10.3389/fphar.2021.649143 (PMC8045977; doi:10.3389/fphar.2021.649143)
Supplement: Supplementary file 7 [file Table3.docx]

### Table A3: GRADE summary for the primary outcomes

| **Intervention** | **Change in MMDs** | **TEAEs** |
| --- | --- | --- |
| **Compared with placebo** |  |  |
| Eptinezumab | High | High |
| Erenumab | High | High |
| Fremanezumab | High | High |
| Galcanezumab | High | High |
| **Compared with eptinezumab** |  |  |
| Erenumab | Low^†§^ | Moderate^§^ |
| Fremanezumab | Moderate | Moderate^§^ |
| Galcanezumab | Moderate^§^ | Moderate^§^ |
| **Compared with erenumab** |  |  |
| Fremanezumab | Moderate^§^ | Moderate^§^ |
| Galcanezumab | Moderate^§^ | Moderate^§^ |
| **Compared with fremanezumab** |  |  |
| Galcanezumab | Moderate^§^ | Moderate^§^ |

MMDs: monthly migraine days; TEAEs: treatment-emerging adverse events

† Rated down for imprecision.

§ Rated down for indirectness
