# Supplementary material for: Efficacy and Safety of Monoclonal Antibody Against Calcitonin Gene-Related Peptide or Its Receptor for Migraine: A Systematic Review and Network Meta-analysis
Source: Front Pharmacol. 2021 Mar 25;12:649143. doi: 10.3389/fphar.2021.649143 (PMC8045977; doi:10.3389/fphar.2021.649143)
Supplement: Supplementary file 8 [file Table4.docx]

| **Intervention** | **MMDs**  **[MD (95%CI)]** | **TEAEs**  **[RR (95%CI)]** |
| --- | --- | --- |
| **Compared with placebo** |  |  |
| Eptinezumab | -1.25 (-2.76, 0.19) | 1.03 (0.86, 1.22) |
| Erenumab | -1.25 (-2.62, 0.13) | 0.90 (0.78, 1.02) |
| Fremanezumab | **-2.05 (-3.32, -0.85)** | 1.08 (0.97, 1.20) |
| Galcanezumab | **-2.28 (-3.22, -1.31)** | 1.07 (0.98, 1.17) |
| **Compared with eptinezumab** |  |  |
| Erenumab | 0.01 (-1.94, 2.06) | 0.87 (0.70, 1.10) |
| Fremanezumab | -0.79 (-2.77, 1.14) | 1.05 (0.86, 1.29) |
| Galcanezumab | -1.02 (-2.73, 0.76) | 1.04 (0.86, 1.27) |
| **Compared with erenumab** |  |  |
| Fremanezumab | -0.81 (-2.69, 1.00) | 1.21 (1.01, 1.43) |
| Galcanezumab | -1.02 (-2.71, 0.67) | 1.19 (1.01, 1.41) |
| **Compared with fremanezumab** |  |  |
| Galcanezumab | -0.22 (-1.76, 1.37) | 0.99 (0.86, 1.14) |

Supplementary table 4. Sensitivity analysis for the primary outcomes.

MMDs: monthly migraine days; TEAEs: treatment-emergent adverse events; MD: mean difference; RR: relative risk; CrI: credibility interval
